# Social media during a pandemic: bridge or burden?

**DOI:** 10.1590/1516-3180.2020.0151.08052020

**Published:** 2020-06-05

**Authors:** Pedro Shiozawa, Ricardo Riyoiti Uchida

**Affiliations:** I PhD. Assistant Professor, Department of Mental Health, Faculdade de Ciências Médicas, Santa Casa de São Paulo (FCMSCSP), São Paulo (SP), Brazil.; II

Dear Editor,

Since the late 2000s, humanity has gradually moved towards accessing the internet with the aim of seeking social media rather than for any other purpose. The web has undoubtedly become empowered over the last decade as a global communication tool.^1^

Many authors have underscored a central issue regarding social media: while networks enable interactions with larger numbers of people, they also inevitably lead towards a reduction in interpersonal communication within the family and within the physical environment.^2^ The decrease in time spent on direct face-to-face interaction can contribute to psychiatric and psychological problems. In fact, recent studies have highlighted that longer times spent on social media can give rise to distorted impressions of oneself and correlate with the intensity of depressive symptoms.^3^

Interestingly, Facebook users tend to perceive others as happier than themselves and, thus, are more likely to express the feeling that “life is not fair”.^4^ This misconception is problematic, since perceiving others as happier and more successful can act as a stressor for mental dysfunction.^1^ A huge paradox has consequently arisen: people are increasingly in contact with one another throughout a myriad of screens and devices, but have never before felt so lonely.

Nonetheless, the boom in social media has also been a bright spot for mental health. Different researchers have shed some light over positive uses of online communication with friends and family, and the main results indicate that online communication is associated with decreased depression.^5^ In other words, use of social media to strengthen preexisting affective bonds seems to function as a factor of social insertion, thereby protecting individuals against mental illnesses. Moreover, different web-based tools focusing on screening for psychiatric symptoms and clinical assessments have been advancing by leaps and bounds.

The harmful potential of social media seems, thus, to depend on people’s own *modus operandi*. In other words, use of computers has been cautiously seen as a useful tool if well used, but has also been seen as a potentially insidious enemy, if used without a suitable purpose and not in keeping with the overall scenario.

Hence, all in all, in relation to the current scenario of the COVID-19 pandemic, the question that arises is the following: Can use of social media aid in overcoming the burden of inevitable isolation?

Regarding the current medical literature, no specific studies with emphasis on social media and pandemics have yet been conducted. However, two main strategies can be derived from data on daily use of social media that are already available. Firstly, use of social media can become a real-time communication strategy for helping data and information to circulate during a pandemic. This follows from the recommendations made in the current COVID-19 pandemic, in which widespread use of cutting-edge information technology to raise awareness about some particular event has been highlighted as a fundamental approach towards dealing with the crisis. Secondly, use of social media can put people side by side if they have been forced apart and have already become stressed, as commonly seen during quarantines. Hence, social media have the ability to bring people closer together when they are unable to physically see each other.

In conclusion, it is not a matter of how much use is made of social media, but rather an issue of the way in which these media and new technologies are used: this is the key point underscoring the usefulness of online and virtual social interactions.
